# Adiponectin and cancer: a systematic review

**DOI:** 10.1038/sj.bjc.6603051

**Published:** 2006-03-28

**Authors:** I Kelesidis, T Kelesidis, C S Mantzoros

**Affiliations:** 1 Division of Endocrinology, Diabetes and Metabolism, Beth Israel Deaconess Medical Center, Boston, MA, USA

**Keywords:** obesity, insulin resistance, adipocytokines, adiponectin

## Abstract

Recent studies have demonstrated that obesity is a significant risk factor for the development of several malignancies, but the mechanisms underlying this relationship remain to be fully elucidated. Adiponectin, an adipocyte secreted endogenous insulin sensitizer, appears to play an important role not only in glucose and lipid metabolism but also in the development and progression of several obesity-related malignancies. In this review, we present recent findings on the association of adiponectin with several malignancies as well as recent data on underlying molecular mechanisms that provide novel insights into the association between obesity and cancer risk. We also identify important research questions that remain unanswered.

Obese subjects have not only increased risk of developing cancer (Wolk *et al*, 2001), but their mortality is also increased with increasing BMI, especially when the BMI is >40 kg m^−2^. More specifically, obesity has been identified as a risk factor for several cancers including endometrial cancer and breast cancer (especially after menopause), colon and rectal, oesophageal, kidney, pancreatic, biliary, ovarian, cervical and liver cancer (Wolk *et al*, 2001; Larsson and Wolk, 2006). Inconsistent data have been reported for prostate cancer (Wolk *et al*, 2001; Larsson and Wolk, 2006) but most studies have suggested a modest increase in risk of advanced prostate cancer with increasing body mass. Although adenocarcinoma of the gastric cardia is obesity related, data are limited and inconsistent for noncardia cancers of the stomach as is on the relationship between haematopoietic malignancies and BMI (Wolk *et al*, 2001; Larsson and Wolk, 2006).

Consequently, there is evidence in humans for a cancer-preventive effect of avoidance of weight gain for cancers of colon, breast (postmenopausal), endometrium, kidney and adenocarcinoma of the esophagus (Wolk *et al*, 2001; Larsson and Wolk, 2006). The above epidemiologic associations and recommendations are consistent with animal studies showing that caloric restriction dramatically decreases spontaneous and carcinogen-induced tumour incidence, multiplicity and size (Wolk *et al*, 2001; Larsson and Wolk, 2006).

## ENDOCRINE SYSTEMS/ADIPONECTIN AS A LINK BETWEEN OBESITY AND CANCER

Understanding the associations between overweight, obesity and a wide variety of cancers, as well as the biological mechanisms contributing to these associations, remains an evolving and currently very active area of research. Steroid hormones, insulin, insulin-like growth factor systems and adipokines are endocrine systems that have been associated with carcinogenesis (Wolk *et al*, 2001). We focus herein on adiponectin; a recently identified adipocyte secreted endogenous insulin sensitizer, circulating levels of which have been associated with insulin resistance/insulinemia and sex steroids.

### Adiponectin: general information

Adiponectin is a recently identified adipocyte secreted hormone expressed exclusively in adipocytes. Also called gelatin-binding protein-28 (GBP28), AdipoQ, ACRP30 or apM1, adiponectin is considered a link between obesity, insulin resistance and diabetes. The human adiponectin gene (apM1) is located on chromosome 3q27 coding for a 244 amino-acid polypeptide (Chandran *et al*, 2003).

Adiponectin consists of three domains including a signal peptide, a collagen-like motif and a globular domain. In addition, adiponectin exists in the circulation in at least two forms, low molecular weight (LMW) oligomers that is hexamers (two trimers) and high molecular weight (HMW) oligomers consisting of four–six trimers. Although the majority of intracellular adiponectin consists of HMW oligomers, LMW oligomers are the predominant form of adiponectin in the circulation (Pajvani *et al*, 2004). It has been recently suggested that the HMW adiponectin complex is the major source of the active form of this protein (Pajvani *et al*, 2004). Adiponectin is abundant in human plasma, with concentrations ranging from 3 to 30 *μ*g ml^−1^ and accounting for up to 0.05% of total plasma protein (Chandran *et al*, 2003).

### Determinants of adiponectin levels in the circulation

In contrast to most adipose-tissue-derived proteins, plasma adiponectin levels are found to be lower in obese than in lean subjects. Thus, a negative correlation between obesity, especially central obesity, insulin resistance, type 2 diabetes (T2D) and circulating adiponectin has been well established (Chandran *et al*, 2003). Adiponectin concentrations increase with weight loss (Chandran *et al*, 2003). Thus, plasma adiponectin levels are reduced in (a) in obese and diabetic mice and humans (Trujillo and Scherer, 2005) (b) cardiovascular disease (Trujillo and Scherer, 2005), (c) hypertension and/or (d) metabolic syndrome (Trujillo and Scherer, 2005). Testosterone decreases adiponectin levels and adiponectin has recently been inversely associated with oestrogen levels. Thus, although women have higher adiponectin levels than men independently of body fat mass or fat distribution (Cnop *et al*, 2003), postmenopausal women have significantly higher adiponectin levels compared with premenopausal women (Cnop *et al*, 2003). In addition, adiponectin gene expression is reversibly downregulated by insulin and plasma adiponectin concentration is inversely correlated with fasting plasma insulin (Hotta *et al*, 2000). Recent studies (Qi *et al*, 2005) suggest that dietary factors may modulate plasma adiponectin concentrations. Caloric restriction and weight loss as well as diets with low glycemic load increase adiponectin levels (Qi *et al*, 2005; Mantzoros *et al*, unpublished observations).

### Adiponectin receptors

Two adiponectin receptor forms (AdipoR1 and R2) have recently been cloned (Yamauchi *et al*, 2003) and shown to have unique distributions and affinities for the different forms of circulating adiponectin. Specifically, AdipoR1 is a high-affinity receptor for globular adiponectin, but also has a very low affinity for the full-length molecule and is expressed ubiquitously. It is most abundant in skeletal muscle, but is also present in endothelial cells and other tissues (Goldstein and Scalia, 2004). AdipoR2 has intermediate affinity for both forms of adiponectin and is predominantly expressed in the liver (Goldstein and Scalia, 2004). AdipoR1 and AdipoR2 appear to be integral membrane proteins; the N terminus is internal, and the C terminus is external, which is opposite to the topology of all other reported G protein-coupled receptors (Yamauchi *et al*, 2003). AdipoR1 and AdipoR2 may form both homo- and heteromultimers, and it is increasingly recognized that adiponectin receptors mediate fatty-acid oxidation and glucose uptake by adiponectin (Yamauchi *et al*, 2003). Finally, T-cadherin, a cell-surface receptor located on endothelial and smooth muscle cells, involved in calcium mediated cell–cell interactions and signalling was also found to bind strongly HMW forms but not trimeric/globular forms of adiponectin and it is thought to act as a coreceptor (Hug *et al*, 2004). The histochemical localization of T-cadherin is intriguing because it is similar to the localization of adiponectin receptors (Hug *et al*, 2004). T-cadherin may participate in signalling through its association with other membrane proteins and incorporation into specific lipid domains of the cell membrane and/or it may restrict proliferation of the intima through its interactions with adiponectin (Hug *et al*, 2004). The significance of similar interactions in the area of carcinogenesis remains to be fully explored.

### Adiponectin and risk of various types of cancer

Our group and others have recently reported that circulating adiponectin levels *in vivo* are inversely associated with the risk of malignancies associated with obesity and insulin resistance (Housa *et al*, 2005), that is, endometrial cancer (Petridou *et al*, 2003; Dal Maso *et al*, 2004), postmenopausal breast cancer (Mantzoros *et al*, 2004), leukaemia (Petridou *et al*, 2006) and colon cancer (Wei *et al*, 2005). Moreover, low adiponectin levels have been associated with gastric cancer (Ishikawa *et al*, 2005) and prostate cancer (Goktas *et al*, 2005).

#### Adiponectin and breast cancer

Obesity has been shown to increase rates of breast cancer in postmenopausal women consistently by 30−50% (Wolk *et al*, 2001). As insulin resistance and obesity have been associated with development of breast cancer, we hypothesized that decreased adiponectin levels might underlie the association between breast cancer and obesity (Mantzoros *et al*, 2004). Our initial observations based on a case control study, were confirmed by subsequent studies showing that lower-circulating adiponectin levels are associated with increased risk of breast cancer (Miyoshi *et al*, 2003; Mantzoros *et al*, 2004; Chen *et al*, 2005) independent of age, menopause status, hormone receptor status, lymph nodes metastases, status of oestrogen receptor, HER2/neu (Chen *et al*, 2005). Additionally, some but not all studies have suggested that breast tumours arising in women with low-serum adiponectin levels may have a more aggressive phenotype (large size of tumour and high histological grade) (Miyoshi *et al*, 2003; Mantzoros *et al*, 2004; Chen *et al*, 2005).

#### Adiponectin and endometrial cancer

In women diagnosed with endometrial cancer, obesity is more prevalent in premenopausal, compared with postmenopausal women (Wolk *et al*, 2001). We were the first to report that adiponectin is inversely related with risk of endometrial cancer, especially among women younger than 65 years (Petridou *et al*, 2003). The inverse relation of adiponectin with risk of endometrial cancer is independent of possible effects of IGF-I, IGF-II, IGFBP-3, leptin, BMI and other known risk factors of the disease, but the combination of high BMI and low adiponectin levels led to a more than six-fold excess risk of endometrial cancer (Miyoshi *et al*, 2003; Mantzoros *et al*, 2004; Petridou *et al*, 2003).

#### Adiponectin and colon cancer

We have also recently evaluated the association between adiponectin and colorectal cancer in a case–control study nested in the large prospective Health Professionals Follow-up study and found that plasma adiponectin levels were inversely associated with risk for colorectal cancer in men (Wei *et al*, 2005). Individuals in the highest adiponectin quintile had an approximately 60% reduced risk for colorectal cancer compared to the lowest quintile, the association being independent of body mass index, waist circumference, waist-to-hip ratio and physical activity (Wei *et al*, 2005).

#### Adiponectin and gastric cancer

Plasma adiponectin levels have also been found to be lower in patients with gastric cancer, especially in upper gastric cancers, compared to normal controls and are inversely correlated with tumour size, depth of invasion and tumour TNM stage, suggesting a potential role for adiponectin in progression of gastric cancer (Ishikawa *et al*, 2005).

#### Adiponectin and prostate cancer

A small case–control study (Goktas *et al*, 2005) indicates that plasma adiponectin levels are significantly lower in subjects with prostate cancer than in subjects with benign prostatic hyperplasia or in normal healthy controls. In addition, plasma adiponectin levels were negatively associated with histological grade and disease stage (Goktas *et al*, 2005).

#### Adiponectin and leukaemia

Adiponectin suppresses the growth of myelomonocyte cells lines, and induces apoptosis in myelomonocytic progenitor cells (leukaemia lines) *in vitro* (Yokota *et al*, 2000). We have recently shown that circulating adiponectin is inversely associated with risk for acute myelogenous leukaemia (AML) but not acute lymphoblastic leukaemia (ALL) in children (Petridou *et al*, 2006).

### Mechanisms that may link adiponectin with carcinogenesis

#### Indirect effects through altering hormone and cytokine levels

Circulating adiponectin concentrations are inversely correlated with fasting plasma insulin (Hotta *et al*, 2000), and adiponectin stimulates the sensitivity of peripheral tissue to insulin (Yamamoto *et al*, 2002). Several studies have supported an association of elevated insulin production and/or insulin concentrations and decreasing levels of IGFBP-3, with an increased risk of developing several malignancies including colorectal and breast cancer (Kaaks *et al*, 2000; Moschos *et al*, 2002). In obesity, reduced adiponectin levels lead to the development of insulin resistance and compensatory, chronic hyperinsulinaemia. Increased insulin levels, in turn, lead to reduced liver synthesis and blood levels of insulin-like growth factor binding protein 1 (IGFBP1) and IGFBP2, and probably also reduce IGFBP1 synthesis locally in other tissues. This results in increased levels of bioavailable IGF1. Insulin and IGF1 signal through the insulin receptors (IRs) and IGF1 receptor (IGF1R), to promote cellular proliferation and inhibit apoptosis in many tissue types upregulating the secretion of vascular endothelial growth factor, contributing thus to carcinogenesis (Calle and Kaaks, 2004).

Adiponectin has also been shown to inhibit both the production of TNF-a in macrophages and its action in endothelial cells (Ouchi *et al*, 1999). Although generally considered proapoptotic, TNF-a can also stimulate both oestrogens biosynthesis and angiogenesis (Rose *et al*, 2004). Therefore, low adiponectin levels can potentially lead to carcinogenesis through altered effect of TNF-a on tumour cell proliferation. Further studies are needed to fully elucidate these and potentially other molecular pathways.

#### Direct effects of adiponectin on carcinogenesis

Adiponectin can also protect from carcinogenesis through more direct effects. Specifically, adiponectin has been found to be an important negative regulator of hematopoiesis and the immune system (Yokota *et al*, 2000). Yokota *et al* have shown that adiponectin suppresses the growth of myelomonocyte cells lines *in vitro*, induces apoptosis in myelomonocytic progenitor cells (leukaemia lines), modulates expression of apoptosis-related genes in M1 cells and downregulates Bcl-2 gene expression (Yokota *et al*, 2000).

Adiponectin selectively binds to several mitogenic growth factors, such as PDGF-BB, basic FGF and HB EGF (Wang *et al*, 2005), that can induce proliferation in many types of cells. The interaction of adiponectin with these growth factors can preclude their binding to the membrane receptors suggesting that the antiproliferative effect of adiponectin could at least in part be due to its selective sequestration of growth factors at a prereceptor level(Wang *et al*, 2005). Moreover, adiponectin may inhibit activation of nuclear factor – *κ*B (NF-*κ*B), a transcription factor that upregulates VEGF in breast cancer cells.

#### Signalling pathways linking adiponectin with carcinogenesis

Several signalling molecules such as 5′-AMP-activated protein kinase (AMPK), nuclear factor-*κ*B (NF-*κ*B), peroxisome proliferators-activated receptor (PPAR)-*α* and p38 mitogen-activated protein (MAP) kinase are known to mediate adiponectin-induced metabolic effects. More recently, c-Jun NH2-terminal kinase (JNK) and signal transducer and activator of transcription 3 (STAT3) were also shown to be downstream effectors of adiponectin (Miyazaki *et al*, 2005).

**Figure 1:**
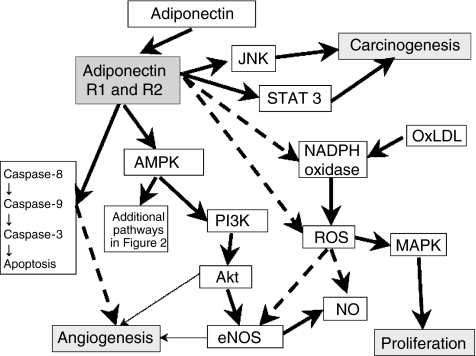
Multiple potential signalling pathways for adiponectin. Abbreviations: R1 and R2, adiponectin receptor type 1 and 2; AMP kinase, adenosine 5′-monophosphate (AMP)-activated protein kinase; NADPH, nicotinamide adenine dinucleotide phosphate; oxLDL, oxidized low-density lipoprotein; PI-3K, phosphatidylinositol-3-kinase; Akt, agarose kinase target or protein kinase B; ROS, reactive oxygen species; NO, nitric oxide; eNOS, endothelial NO synthase; MAPK, mitogen-activated-protein-kinase. The solid arrows and dotted lines reflect stimulatory and inhibitory effects, respectively. Modified from Goldstein and Scalia: JCEM, June 2004, 89(6):2565.

**Figure 2:**
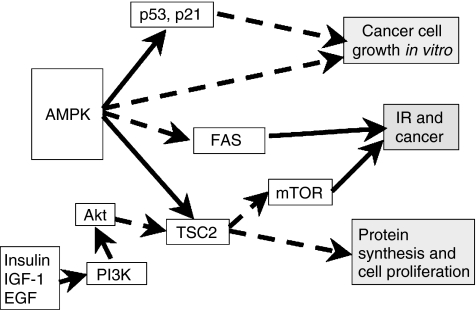
Possible molecular mechanisms of regulation of tumour cell growth by AMPK (12). Abbreviations: AMP kinase, adenosine 5′-monophosphate (AMP)-activated protein kinase; PI-3K, phosphatidylinositol-3-kinase; Akt, agarose kinase target or protein kinase B; mammalian target of rapamycin (mTOR); fatty acid synthase (FAS). The solid arrows and dotted lines reflect stimulatory and inhibitory effects, respectively.

*(a) AMPK, adiponectin*, *and their association with carcinogenesis* (Figures 1 and 2): The molecular pathways downstream of AdipoR remain to be fully elucidated, but studies in metabolically responsive cells have shown that activation of the pleiotropic AMPK is part of the signalling cascade downstream of adiponectin receptor (Goldstein and Scalia, 2004; Luo *et al*, 2005). AMPK is a ubiquitously expressed *αβγ* heterotrimer consisting of a catalytic subunit (*α*) and two noncatalytic subunits (*β*) (*γ*). In addition to exercise and starvation, AMPK is activated by the fat-cell-derived hormones adiponectin and leptin (Yamauchi *et al*, 2002), catecholamines (Kelly *et al*, 2004) and IL-6 (Kelly *et al*, 2004). Once activated, AMPK exerts direct effects on specific enzymes and transcriptional regulators, stimulating multiple events that enhance ATP generation and inhibiting others that consume ATP but are not acutely necessary for survival (Luo *et al*, 2005). The enzymes downstream of AMPK include the mammalian homologue of target of rapamycin (mTOR), acetyl-CoA carboxylase (ACC), fatty acid synthase (FAS) and glycerol phosphate acyltransferase (GPAT), which are key regulators of protein, fatty acid and glycerolipid synthesis, respectively (Luo *et al*, 2005).

AMPK has been reported to inhibit FAS (Kuhajda, 2000; Luo *et al*, 2005), a key lipogenic enzyme, which has been associated with colon, breast, prostate and ovarian cancer (Kuhajda, 2000). Finally, AMPK phosphorylates and activates TSC2, a tumour suppressor that negatively regulates protein synthesis by inhibiting mTOR. mTOR has been associated with colon, breast, prostate, ovarian, liver and lung cancer (Philp *et al*, 2001). The regulation of TSC2 and mTOR by AMPK might have special implications because the PI3K-Akt signalling pathway is constitutively active in many cancers (Figure 2). The activation of this pathway is most notable in cancers that have either inactivating mutations of the PTEN gene (e.g. glioblastoma, melanoma, prostate cancer and endometrial carcinomas) or overexpression of an activated member of the epidermal growth factor (EGF) receptor family (e.g. Her2/neu in breast cancer) (Luo *et al*, 2005).

Activation of AMPK by adiponectin also activates endothelial NO synthase (eNOS) and increases nitric oxide (NO) production. eNOS activation is also dependent on signalling through Akt kinase (Akt) and its upstream mediator phosphatidylinositol 3-kinase (PI-3K) (Goldstein and Scalia, 2004). The exact role of the adiponectin pathway in the inhibition of angiogenesis remains to be fully elucidated. As AMPK may also stimulate angiogenesis *in vitro* in response to hypoxia (Ouchi *et al*, 2005), whether an antiangiogenic effect of adiponectin is mediated by AMPK or eNOS-NO needs to be further investigated.

In summary, through these actions AMPK might reduce the risk for developing cancers for which molecules such as mTOR may act like mitogens (Luo *et al*, 2005) (Figure 2). As adiponectin stimulates AMPK, (Goldstein and Scalia, 2004; Luo *et al*, 2005) which might inhibit the growth and/or survival of cancer cells, (Saitoh *et al*, 2004) the above molecular pathways may be potential mechanisms through which adiponectin regulates carcinogenesis.

*(b) Other mechanisms (independent of AMPK) linking adiponectin with carcinogenesis:* Adiponectin inhibits superoxide production (ROS), possibly through inhibition of cellular NADPH oxidase activity (Goldstein and Scalia, 2004). Reduced ROS generation may increase NO production (by improvement of the suppression of eNOS activity by ROS) and reduce cell proliferation by blocking oxLDL induced mitogen-activated protein kinase pathway (MAPK) activation (Goldstein and Scalia, 2004) (Figure 1).

Finally, adiponectin may also regulate angiogenesis negatively (independently of AMPK) through induction of apoptosis in vascular endothelial cells by activating the caspase cascade, a group of apoptotic enzymes. Adiponectin initially activates caspase-8, which subsequently leads to activation of caspases-9 and -3, which in turn leads to cell death (Brakenhielm *et al*, 2004).

*(c) JNK and STAT 3: a novel-signalling pathway linking adiponectin with carcinogenesis* (Figure 1): JNK belongs to the mammalian mitogen-activated protein (MAP) kinase families, is activated in response to various stimuli such as cytokines, and mediates the phosphorylation and activation of transcription factors such as c-Jun (Davis, 2000). In addition to its role in obesity and insulin resistance, JNK is involved in the regulation of cell proliferation and apoptosis during various physiological and pathological events, including tumour development (Davis, 2000). The transcription factor STAT3 also regulates cellular function such as cell proliferation, survival, differentiation and apoptosis (Bowman *et al*, 2000). STAT3 is activated by adipokines such as IL-6, leptin, which activate the Janus kinase-signal transducer and activator of transcription (JAK-STAT) pathway. Dysregulation of the STAT system directly contributes to malignant transformation and cancer progression (Bowman *et al*, 2000). It was recently demonstrated that adiponectin stimulates JNK activation in prostate cancer (DU145, PC-3 and LNCaP-FGC cells), hepatocellular carcinoma (HepG2) cells and C2C12 myoblasts, but also drastically suppresses STAT3 activation in DU145 and HepG2 cells, in which STAT3 is constitutively activated (Miyazaki *et al*, 2005). This suggests that adiponectin may affect the pathogenesis of prostate and hepatocellular carcinomas, which express AdipoR1 and R2 receptors, by acting on tumour cells directly through modulation of JNK and STAT3 (Miyazaki *et al*, 2005). More studies are needed to further elucidate this area.

## CONCLUSION

Adipose tissue is no longer considered an inert depot storage organ but an active endocrine organ. Among the various proteins released by adipocytes, adiponectin appears to play an important role not only in glucose and lipid metabolism but also in the development and progression of various types of cancers as well. Low serum adiponectin levels may be a novel risk factor for cancer and study of adiponectin biology can provide new insights into the association of obesity with cancer risk. Since the mechanisms of action of adiponectin are not entirely clear, future studies are needed to fully elucidate the action of this hormone.

Accumulating evidence indicates that adiponectin measurements may serve as a useful screening tool for predicting risk for, and/or for early detection of obesity related cancers. Adiponectin *per se* or adiponectin analogues may prove to be effective anticancer agents and may have important therapeutic implications. In addition, methods to increase circulating adiponectin levels including PPAR*γ* agonists or methods leading to upregulation of adiponectin receptors and/or development of specific adiponectin receptor agonists (e.g. osmotin) (Kadowaki and Yamauchi, 2005), could prove beneficial in several obesity related malignancies. In this regard, more intensive basic and clinical research efforts are warranted.
